# The Pharmacological Management of Essential Tremor and Its Long-Term Effects on Patient Quality of Life: A Systematic Review

**DOI:** 10.7759/cureus.76016

**Published:** 2024-12-19

**Authors:** Oqab Alharbi, Sofian A Albaibi, Abdullah A Almutairi, Emad Alsaqabi, Meshal Alharbi, Bader S Alharbi, Mohammad F Almansour, Zainah A Al-Qahtani

**Affiliations:** 1 Neurology, Unaizah College of Medicine and Medical Sciences, Qassim University, Unaizah, SAU; 2 Medicine, Unaizah College of Medicine and Medical Sciences, Qassim University, Unaizah, SAU; 3 Internal Medicine, Unaizah College of Medicine and Medical Sciences, Qassim University, Unaizah, SAU; 4 Neurology, King Khalid University, Abha, SAU

**Keywords:** deep brain stimulation (dbs), emerging therapies, essential tremor (et), pharmacological management, quality of life (qol)

## Abstract

Essential tremor (ET) is one of the most prevalent nerve-related movement disorders, most commonly affecting the hands during voluntary movements or while maintaining posture. Unlike tremors in neurodegenerative conditions, ET is not observed at rest. Continued research is essential to optimize treatment strategies and address the unmet need for sustainable, patient-centered therapies that minimize side effects and enhance long-term quality of life (QoL) for individuals with ET. Five medical databases were searched for content relevant to this study’s topic, utilizing the guidelines set forth by Preferred Reporting Items for Systematic Reviews and Meta-Analyses (PRISMA). These databases searched include Web of Science, PubMed, Cochrane Library, ScienceDirect, and Google Scholar for publications between 2010 and 2024. The review analyzed studies including adult patients with ET, focusing on efficacy, safety, and QoL outcomes, with priority given to studies from the Middle East or Saudi Arabia. We included 10 studies that met our inclusion criteria for a full review. Based on the studies, pharmacological treatments such as alprazolam, primidone, propranolol, and CX-8998 were found to be efficient in the management of ET. Emerging medications, including CX-8998 and alprazolam, showed mixed results with significant adverse events. Surgical interventions like deep brain stimulation (DBS) demonstrated long-term motor control benefits, although functional declines occurred due to disease progression. Novel approaches like low-intensity focused ultrasound (LIFU), transcutaneous afferent patterned stimulation (TAPS), and incobotulinumtoxinA injections presented promising results with improved tremor control and minimal side effects. In conclusion, pharmacological treatments for ET provide symptomatic relief but are limited by side effects and reduced long-term efficacy, significantly impacting QoL. Surgical and novel therapeutic options offer enhanced motor control and durability of effects, though they are not universally applicable.

## Introduction and background

Essential tremor (ET) is a common neurological movement disorder marked by involuntary rhythmic shaking, primarily in the hands, though it can also affect the arms, head, voice, and other body parts [1]. ET is a syndrome marked by isolated tremors in both upper limbs, persisting for at least three years, which may also extend to areas like the head, voice, or lower limbs. “Isolated” tremor refers to cases where tremor is the single noticeable sign, distinguishing it from “combined” tremor, which includes additional neurological symptoms. “Essential tremor plus” describes cases with minor neurological signs, such as mild gait abnormalities or dystonic postures, that do not qualify for separate diagnoses [2-4].

ET is unlike tremors associated with other neurodegenerative disorders like Parkinson’s disease - ET occurs mainly during voluntary movements or while maintaining a posture rather than at rest [1-4]. While not life-threatening, ET significantly affects a patient’s quality of life (QoL) by impacting daily activities such as writing, eating, and personal care [1,2,4,5]. ET is fairly prevalent and affects about 0.9% of the global population [2]. The onset of ET remains uncertain, though studies have shown that it may emerge at any age from 15 to 70 years [2-5]. ET is progressive, often worsening gradually over time, and is particularly associated with additional physical and cognitive health issues among those diagnosed later in life, typically after age 65. The prevalence of ET is not gender-specific and may occur to anyone at any time [1,2,6].

The exact cause of ET remains unclear, though mild neurodegeneration in the cerebellum - the brain region responsible for movement coordination - has been suggested as a contributing factor [2,4,6]. Genetics are thought to play a role in ET prevalence, where a strong familial connection has been drawn among patients. ET may occur independently or comorbid to other neurological conditions such as stroke, Parkinson’s disease, and multiple sclerosis [7]. Stress, certain medications like beta-blockers and anticonvulsants, thyroid disorders, and systemic illnesses, including diabetes and kidney failure, may exacerbate symptoms [7]. The majority of ET cases present with mild and stable symptoms that progressively worsen over time.

ET is diagnosed based on a physical examination and a thorough review of the individual's medical history, as well as a neurological examination to test muscle tone and strength, reflexes, balance, and speech [8]. Physicians may also take blood or urine samples to rule out other contributing factors. Diagnostic imaging of the brain is also carried out to assess brain damage, and an electromyogram to assess involuntary muscle movement and response to nerve stimulation [2,5,8]. Pharmacological treatment options are often pursued to manage symptoms with a view to improving a patient’s functionality and comfort while going by their daily routine.

Despite significant global research on ET, a notable gap persists, particularly in the Kingdom of Saudi Arabia, regarding the pharmacological management of ET and its long-term effects on patient QoL. This systematic review, therefore, seeks to address this gap by providing a comprehensive systematic review of existing pharmacological ET treatments.

## Review

Materials and methods

Literature Search Strategy

Five medical databases were searched for content relevant to this study’s topic, utilizing the guidelines set forth by Preferred Reporting Items for Systematic Reviews and Meta-Analyses (PRISMA). These databases include Web of Science, PubMed, Cochrane Library, ScienceDirect, and Google Scholar for publications between 2010 and 2024. A series of keywords was utilized and tailored to each database. The keywords included “tremors”, “essential tremor”, “neurodegenerative”, “neurodegenerative disease”, “beta-blockers”, “anticonvulsant”, “action tremor”, “tremor syndrome”, “progressive essential tremor”, and “upper limb tremor management”. 

Eligibility, Data Extraction, and Management

Rayyan (Qatar Computing Research Institute, Ar-Rayya, Qatar) tool was utilized to rigorously assess the retrieved articles for eligibility, ensuring they met the pre-established inclusion and exclusion criteria defined collaboratively by all researchers involved in the study.

Inclusion and Exclusion Criteria

The inclusion criteria for this review focused on studies published from 2010 onward in English and with strong study designs, including randomized controlled trials, observational studies, systematic reviews, and meta-analyses. Studies selected involved adult populations diagnosed with ET, those under pharmacological treatments such as beta-blockers, anticonvulsants, or benzodiazepines. Eligible studies were required to report on treatment efficacy, safety, or QoL outcomes. Studies from the Middle East or Saudi Arabia were given priority to enhance regional relevance.

Exclusion criteria eliminated studies published before 2010 or in languages other than English. Studies focusing on pediatric populations, animal models, or non-pharmacological interventions were also excluded. Studies whose research primarily centered on other neurological disorders were not considered unless ET was the primary focus. Case reports, editorials, letters to the editor, and articles lacking explicit treatment outcomes were excluded from the review. The inclusion and exclusion criteria were designed to ensure that only high-quality, relevant studies were included in this review. PRISMA guidelines were carefully followed throughout. Any disagreements during the research process were resolved through thorough, collaborative discussions to reach a consensus.

Statistical Data Analysis

We used RevMan version 5.4.1 (The Cochrane Collaboration, Oxford, UK) software for data analysis. The statistical approach focused on assessing the quality and risk of bias across included studies. For retrospective studies, the Newcastle Ottawa Quality Assessment Scale was applied, rating studies based on selection, comparability, and outcomes. The funnel plot accessed the publication bias.

Results

**Figure 1:**
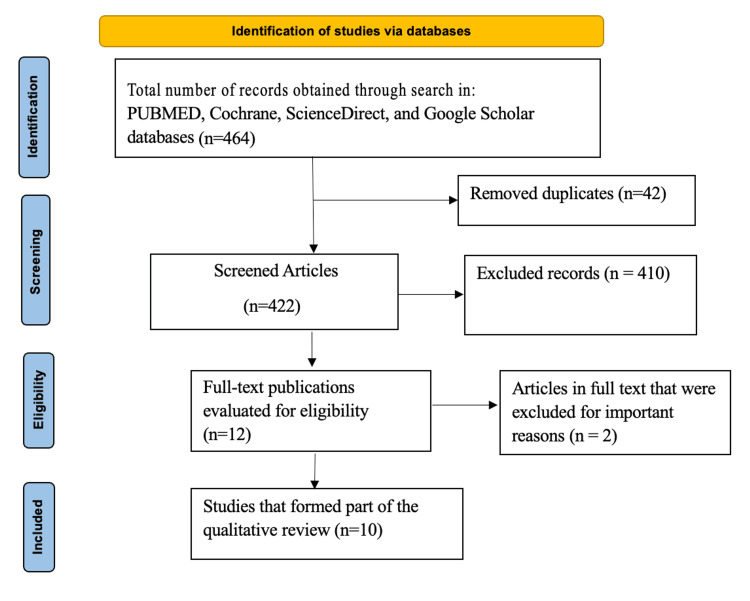
Preferred Reporting Items for Systematic Reviews and Meta-Analyses (PRISMA) flow diagram

There were 464 articles found in the initial search from the various databases: Web of Science (21), PubMed (299), Cochrane Library (9), ScienceDirect (92), and Google Scholar (43). The studies were screened through the above eligibility criteria, with a final number of 10 studies eligible for inclusion and analysis as illustrated in Figure 1 below:

Study Characteristics

The essential characteristics of the studies included in this review are outlined in Table 1. All the studies were conducted in different regions globally and were published in English.

**Table 1:** Characteristics of the included studies

Authors	Study type	Intervention	Sample size	Outcome/results	Conclusion
Bruno et al., 2015 [9]	RCT	Alprazolam vs. placebo for the treatment of essential tremor (ET).	24 patients	Alprazolam treatment resulted in a notable decrease in tremor severity (MD -0.75, 95% CI -0.83 to -0.67). Adverse effects were reported in 75% of participants receiving alprazolam, with sedation being the most common (50%), followed by constipation (17%) and dry mouth (9%). Neither treatment nor control groups experienced any participant dropouts.	Evidence is insufficient to draw firm conclusions about alprazolam’s efficacy and safety for ET due to the small sample size and methodological limitations. Further research is needed to confirm efficacy and assess long-term safety.
Vetterick et al., 2022 [10]	Retrospective observational study	Analysis of US claims data to examine ET diagnoses, treatment patterns, and adherence from 2015 to 2019.	1336,183 patients with ET diagnosis codes	In 2019, an estimated 213,772 confirmed cases of ET were identified. Among these, 96% had at least one comorbidity, and 64% were treated with pharmacological interventions. The most frequently prescribed medications were propranolol (24%) and primidone (20%). However, nearly 40% of patients discontinued their medications within two years.	The study highlighted gaps in ET care, with approximately one million untreated patients in the US. Challenges include delayed diagnoses, high comorbidity rates, and limited treatment adherence due to tolerability issues. Improved therapies and management strategies are needed.
Deveney et al., 2024 [11]	Pilot open-label clinical trial	Low-intensity focused ultrasound (LIFU) targeting the ventral intermediate nucleus of the thalamus (Vim) for ET treatment.	10 participants	No adverse effects were observed. Eight participants achieved a Global Rating of Change (GRC) score of ≥2, and all participants demonstrated clinically meaningful improvement on the Tremor Research Group Essential Tremor Rating Scale (TETRAS) performance subscale.	Preliminary results indicate that LIFU is safe, feasible, and shows promising efficacy for treating ET. Further sham-controlled studies are needed to confirm these findings.
Tam et al., 2017 [12]	Clinical trial	Use of a magnetic resonance-compatible tablet for quantitative tremor measurement during transcranial magnetic resonance-guided focused ultrasound (MRgFUS) treatment of ET.	12 patients	Post-operative tremor severity in the treated (dominant) hand showed a significant reduction compared to pre-operative levels. However, no substantial changes were seen in the tremor severity of the untreated (non-dominant) hand. Intra-operative tremor measurements fell between the pre- and post-operative values.	The study demonstrated the feasibility and effectiveness of using a computerized tablet system for quantitative tremor measurement during MRgFUS treatment.
Papapetropoulos et al., 2021 [13]	Phase 2 proof-of-concept, randomized, placebo-controlled trial	Evaluation of CX-8998, a selective T-type calcium channel modulator, for the treatment of moderate to severe ET.	95 patients (48 received CX-8998; 47 received placebo)	There was no significant difference in the primary endpoint (video-rated TETRAS-PS scores) between CX-8998 and placebo (P = 0.696). However, investigator-rated TETRAS-PS scores showed significant improvement with CX-8998 (P = 0.017). Additionally, TETRAS-activities of daily living (ADL) and total scores significantly improved with CX-8998. Common side effects included dizziness (21%), headache (8%), euphoria (6%), and insomnia (6%).	CX-8998 improved some measures of ET severity but did not meet the primary endpoint. The results support further investigation to evaluate its potential efficacy and safety.
Bai et al., 2022 [14]	Longitudinal study	Evaluation of the long-term efficacy and predictive factors of ventral intermediate nucleus deep brain stimulation (Vim-DBS) for ET.	533 patients from 18 studies	Significant improvement in motor scores maintained long-term (≥4 years). Reduction in hand function and ADL scores attributed to DBS tolerance and disease progression. Stimulation frequency and preoperative scores were predictive of long-term outcomes. Tremor Rating Scale (TRS) total scores worsened over time due to disease progression, but motor control remained stable.	Vim-DBS provides sustained motor symptom control in ET patients but loses efficacy for hand function and ADL over time due to DBS tolerance and disease progression.
Vogelnik, et al., 2024 [15]	Prospective observational study	Investigation of primidone and propranolol's mechanisms of action in ET using transcranial magnetic stimulation (TMS) and eyeblink classical conditioning (EBCC).	54 patients (28 treated with primidone, 26 with propranolol)	Primidone reduced hand tremor severity, increased corticospinal excitability thresholds, and modulated GABAergic circuits. Propranolol reduced tremor severity via noradrenergic modulation of gamma-aminobutyric acid (GABA) circuits. Better EBCC at baseline predicted a stronger response to primidone, but not propranolol.	Primidone and propranolol demonstrated distinct central mechanisms for alleviating tremor. Primidone's efficacy was linked to its GABAergic effects, while propranolol showed more modest central modulation.
Lowell et al., 2019 [16]	Randomized, double-blind, placebo-controlled, cross-over study	Octanoic acid (OA) treatment for essential voice tremor (EVT).	16 participants	Significant reduction in amplitude tremor magnitude (4.14% with OA vs. 0.48% with placebo, P < 0.05). - Frequency tremor also significantly reduced (1.21% with OA vs. 0.22% with placebo, P < 0.05). No significant differences in auditory-perceptual ratings or self-reported voice disability (Voice Handicap Index-10). Adverse events were mild and similar across both OA and placebo groups.	OA reduced tremor severity objectively but showed no significant impact on perceived voice disability or perceptual ratings. Further studies are needed to optimize dosing and explore its functional impact on communication.
Jog et al., 2020 [17]	Randomized, double-blind, placebo-controlled exploratory trial	Customized intramuscular injections of incobotulinumtoxinA for upper-limb ET, guided by kinematic tremor analysis.	30 patients	Statistically significant improvement in Fahn-Tolosa-Marin (FTM) motor performance scores at weeks 4 (P = 0.003) and 8 (P = 0.031) with incobotulinumtoxinA vs. placebo. Reduced hand-tremor amplitude (P = 0.004 at week 4, P < 0.001 at week 8), with effects lasting up to 24 weeks. Mild, transient adverse events, including localized muscular weakness and grip strength reduction.	Customized incobotulinumtoxinA injections demonstrated safety, tolerability, and efficacy in reducing tremor severity and improving motor function in ET patients.
Dai et al., 2023 [18]	Randomized pragmatic clinical trial	Evaluation of transcutaneous afferent patterned stimulation (TAPS) therapy added to standard of care (SOC) vs. SOC alone for managing ET.	310 patients	Tremor power significantly reduced in the TAPS group compared to SOC (P < 0.0001). Greater improvement in Bain and Findley ADL (BF-ADL) scores in the TAPS group vs. SOC (P = 0.0187). 45% of TAPS group achieved ≥50% tremor power reduction, with 23% achieving ≥70% reduction. Mild, transient adverse events were reported with no serious device-related events.	Adding TAPS therapy to SOC improves tremor power and functional outcomes significantly compared to SOC alone over one month of home use. Results support TAPS as a safe and effective non-invasive option for ET treatment.

Risk of Bias Assessment

The Newcastle Ottawa Scale Quality Assessment Scale instrument was used to assess the risk of bias in both the nonrandomized and observational studies. This scale was used to judge quality in three investigations. One was rated as a high-quality paper, obtaining eight stars [14]; another was of moderate quality, receiving seven stars [15]; and the final study was low quality, receiving four stars [10] (see Table 2).

**Table 2:** Summary of critical evaluation of included retrospective/prospective observational studies using the Newcastle Ottawa Quality Assessment Scale methodology A study could obtain one star (*) for each numbered item in the selection and outcome categories. However, comparability obtained a rating of up to two stars. Based on the assessment findings (**). For selection and outcome, * indicates a low probability of bias. However, for comparability, ** indicates a low chance of bias, whereas (-) indicates a significant risk of bias.

Author	Selection				Comparability	Outcome			Risk of bias
	Representativeness of the exposed cohort	Selection of the non-exposed cohort	Ascertainment of exposure	Demonstration that outcome of interest was not present at the start of the study	Comparability of cohorts on the basis of the design or analysis	Assessment of outcome	Was follow-up long enough for outcomes to occur	Adequacy of follow-up of cohorts	
Vetterick et al., 2022 [10]	*	*	-	*	*	*	-	*	High risk
Bai et al., 2022 [14]	*	*	*	*	**	*	*	*	Low risk
Vogelnik, et al., 2024 [15]	*	*	*	*	**	*	*	-	Moderate risk

**Figure 2:**
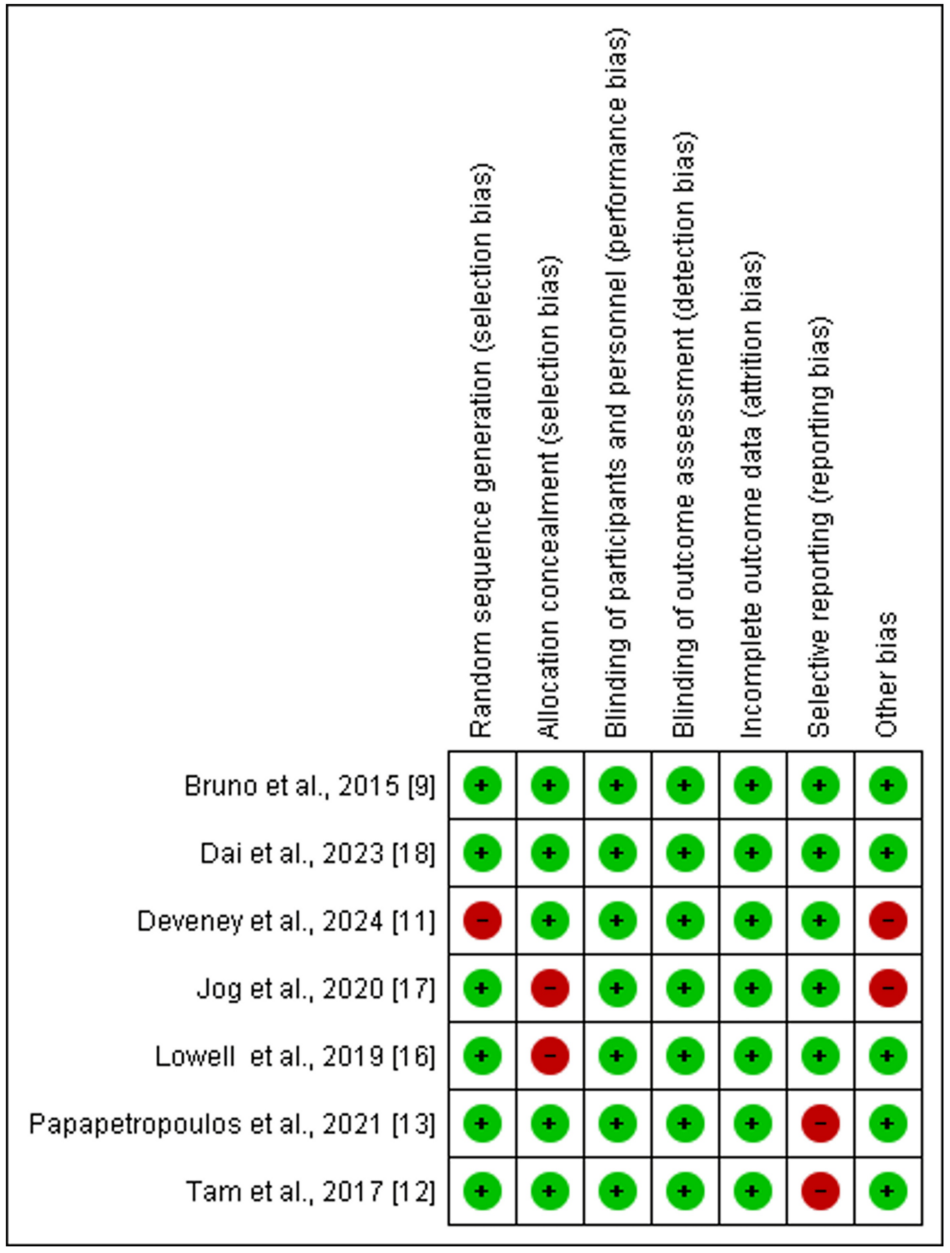
Risk of bias assessment for included studies across seven methodological domains, with green indicating low risk, red indicating high risk, and yellow indicating unclear risk Data from studies [8,11,12,13,16-18].

**Figure 3:**
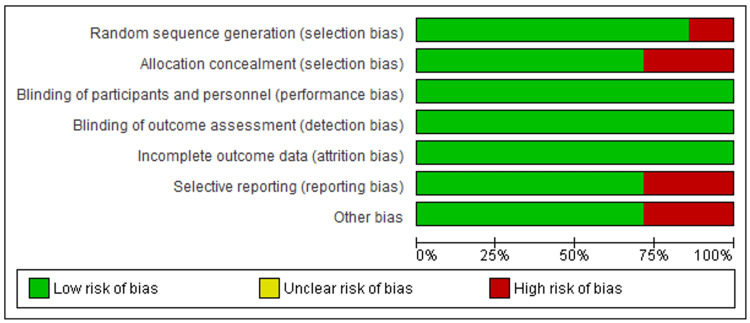
Methodological quality Data from studies [8,11,12,13,16-18].

Figure 2 and Figure 3 below show that 5/7 studies had a low risk of bias [9,12,13,16,18], with all studies scoring six and above out of the seven items assessed for bias. On the other hand, two studies had a moderate risk of bias, scoring five out of seven items used to assess the risk of bias [11,17]. Generally, the included studies were of good quality.

**Figure 4:**
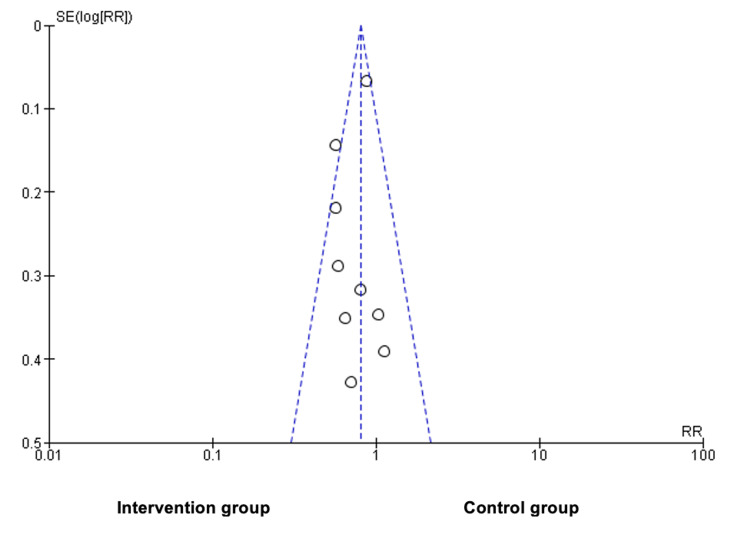
Risk of publication bias SE - standard error, RR - risk ratio

Figure 4 illustrates an asymmetric funnel plot, indicating potential publication bias. The greater concentration of studies on the left side compared to the right suggests an overrepresentation of studies favoring the intervention group. This asymmetry may reflect selective reporting of smaller studies with significant results, potentially skewing the overall findings. Additionally, the presence of an outlier further supports the likelihood of bias or variability in study quality or effect sizes.

Discussion

This systematic review assesses the long-term impacts of pharmaceutical treatments for ET on the QoL of patients. The included studies offer a thorough analysis of the efficacy of several treatments for ET management, emphasizing both tremor reduction and the unpleasant effects that go along with it. Bruno et al. found that alprazolam significantly decreased the severity of tremors (MD -0.75, 95% CI -0.83 to -0.67), although 75% of patients had side effects, including drowsiness (50%), constipation (17%), and dry mouth (9%). Interestingly, neither the treatment nor control groups experienced any dropouts, suggesting that the side effects, while frequent, did not cause cessation [9].

Vetterick et al. reported that 64% of a large cohort of ET patients received pharmacological treatment, with primidone and propranolol being the most commonly prescribed medications. However, approximately 40% of these patients discontinued their treatment within two years, highlighting challenges with long-term adherence and the limited efficacy of these medications in managing ET symptoms [10]. Similarly, Deveney et al., using the Tremor Research Group Essential Tremor Rating Scale (TETRAS), observed significant improvements in tremor severity among treated individuals, with no reported side effects. These findings underscore the treatment's effectiveness and its favorable safety profile, particularly in demonstrating clinically meaningful improvements in the performance subscale [11].

Tam et al. examined the impact of surgical intervention on tremor severity and found significant post-operative improvement in the dominant hand, while the non-dominant hand showed no noticeable changes. These findings suggest that surgical treatments can provide tailored benefits, particularly for the dominant hand [12]. Similarly, Papapetropoulos et al. evaluated the effects of CX-8998, a selective T-type calcium channel modulator, was evaluated in a phase 2 randomized, placebo-controlled trial for moderate to severe ET. The study did not demonstrate significant improvement in the primary endpoint compared to placebo (P = 0.696). However, CX-8998 significantly improved investigator-rated TETRAS-PS scores (P = 0.017) as well as measures of tremor severity and daily activities (TETRAS-activities of daily living (ADL)). Common side effects, including headache, euphoria, dizziness, and insomnia, were moderate in severity [13].

According to Bai et al., patients receiving deep brain stimulation (DBS) saw long-term gains in their motor scores, but as the disease progressed, the severity of their tremors gradually worsened, and their ability to do daily tasks decreased. Although the development of ET may restrict its influence on day-to-day functioning, long-term stability in motor control suggests that DBS can offer long-lasting advantages [14]. Vogelnik Žakelj et al. discovered that by altering distinct brain circuits, primidone and propranolol successfully decreased the severity of tremors. Patients with higher baseline corticospinal excitability responded best to primidone, whereas propranolol helped to modulate GABAergic circuits [15].

In comparison to a placebo, Lowell et al.'s study on the effects of onabotulinumtoxinA (OA) on tremor severity revealed a substantial decrease in tremor frequency and amplitude. Vocal disability and auditory-perceptual evaluations did not significantly improve, though, suggesting that although OA was successful in lowering tremors, it did not treat other ET symptoms. The OA and placebo groups experienced similar and mild adverse effects [16]. According to Jog et al., incobotulinumtoxinA considerably decreased hand tremor amplitude and enhanced Fahn-Tolosa-Marin (FTM) motor scores at four and eight weeks, hence improving tremor severity. Although minor and temporary adverse effects like localized muscle weakness and a decrease in grip strength were observed, these effects lasted for up to 24 weeks [17]. Lastly, Dai et al. discovered that transcutaneous afferent patterned stimulation (TAPS) improved activities of daily living scores more than standard of care (SOC) while also dramatically reducing tremor power. Of the patients in the TAPS group, 23% had a reduction of more than 70% in tremor power, and 45% had at least a 50% reduction. The study noted that TAPS may be a potential therapy option with good safety results, as evidenced by the mild and temporary adverse effects that were observed and the lack of major device-related problems [18].

Some of the limitations of this study are the variability in study designs and patient demographics in the different studies used. The heterogeneity in sample sizes, treatment regimens, and outcome measures makes it difficult to make direct comparisons and limits the generalizability of the findings. Additionally, there was an underrepresentation of studies from specific regions, such as the Middle East, which may not fully capture regional variations in ET management and patient outcomes.

## Conclusions

This review established a variety of pharmacological and emerging therapeutic approaches for managing ET. Some medications like propranolol and primidone remain very important in the ET treatment, but their long-term efficacy is limited by tolerability and adherence challenges. Surgical options like DBS and innovative treatments, including low-intensity focused ultrasound (LIFU) and TAPS, demonstrate promising outcomes with reduced adverse effects. The results highlight the need for customized treatment programs and the necessity for additional research to improve and enhance therapy options for ET patients.
